# Oncocytic Carcinoma: A Rare Hormone-Producing Tumor

**DOI:** 10.7759/cureus.90450

**Published:** 2025-08-18

**Authors:** Francisco Guimarães, Joana Simões, Luísa Fontes, Daniela Filipa Dias, Nuno Pinheiro

**Affiliations:** 1 Internal Medicine, Hospital CUF (Companhia União Fabril) Descobertas, Lisbon, PRT; 2 Endocrinology and Diabetes, Hospital CUF (Companhia União Fabril) Descobertas, Lisbon, PRT; 3 General Surgery, Hospital CUF (Companhia União Fabril) Descobertas, Lisbon, PRT

**Keywords:** adrenal gland neoplasms, diagnostic and therapeutic challenge, endocrine, hypertension-malignant, s: adrenocortical carcinoma (acc)

## Abstract

Oncocytic adrenocortical carcinoma (OACC) is a rare type of malignancy that affects the adrenal cortex. We describe the case of a 68‑year‑old woman with months of refractory hypertension, hypokalemia, headaches, night sweats, and palpitations. Computed tomography revealed a 113 mm left adrenal mass displacing the pancreatic tail and spleen. After a multidisciplinary review, she underwent open adrenalectomy. The 355 g tumor was well circumscribed; histology showed abundant eosinophilic oncocytes arranged in nests and trabeculae, 36 mitoses per 50 high‑power fields (including atypical figures), necrosis, and venous invasion, and the Ki‑67 index was 10%. These findings satisfied major and minor Lin-Weiss-Bisceglia criteria, confirming OACC. Immunohistochemistry was positive for calretinin, Melan‑A, synaptophysin, and inhibin, consistent with adrenocortical origin. Postoperatively, hypertension and potassium levels normalized with reduced antihypertensive therapy; she commenced steroid replacement and adjuvant mitotane. At early follow‑up, she remained clinically stable and disease‑free. This case highlights the importance of considering OACC in patients with large adrenal masses and resistant hypertension, demonstrates the value of multidisciplinary management and definitive surgery, and contributes to the limited evidence guiding adjuvant therapy for this uncommon tumor.

## Introduction

Adrenocortical neoplasms are rare tumors of the adrenal cortex that include oncocytomas, oncocytic neoplasms of uncertain malignant potential, and oncocytic carcinomas. These tumors are rare, with only a few hundred cases reported worldwide and an estimated incidence of 0.5 to 2 cases per million individuals annually [1].

Oncocytic adrenocortical carcinoma (OACC) carries a poor prognosis, in part due to its typically advanced stage at diagnosis. Even after surgical resection, the five-year survival rate ranges from 30% to 50% [2]. When metastatic disease is present at diagnosis, median survival is less than one year [3]. Surgical resection with curative intent remains the primary treatment approach. Although postoperative systemic therapy is often recommended, robust data supporting its efficacy are lacking.

OACC is a rare subset of oncocytic neoplasms. A recent systematic review identified only 83 reported cases worldwide [4]. Oncocytic neoplasms can occur in various organs, including the thyroid, parathyroid glands, kidneys, pituitary gland, salivary glands, and adrenal cortex. These tumors are characterized by the presence of oncocytes - large polygonal cells with prominent granular eosinophilic cytoplasm due to an abundance of cytoplasmic mitochondria [5].

Bisceglia et al. [6] established the modified Lin-Weiss-Bisceglia (LWB) criteria for classifying oncocytic adrenocortical tumors. According to these criteria, a diagnosis of carcinoma requires the presence of at least one major criterion: mitotic rate greater than 5 per 50 high-power fields (HPFs), atypical mitotic figures, or venous invasion. Tumors that meet one or more minor criteria (size >10 cm or weight >200 g, microscopic necrosis, or capsular or sinusoidal invasion) are considered to have borderline malignant potential [6]. Given their rarity, detailed case reports are crucial for building a stronger evidence base and informing future clinical decision-making. We present the case of a 68-year-old woman with severe hypertension, hypokalemia, and an adrenal mass ultimately diagnosed as OACC, highlighting the clinical and pathological features of this rare malignancy.

## Case presentation

A 68-year-old woman with a medical history of well-controlled hypertension and dyslipidemia was treated with chlortalidone 50 mg and candesartan 32 mg daily. Over the preceding three months, she experienced increasingly uncontrolled hypertension, requiring multiple clinical visits for medication adjustments without improvement. This was accompanied by headaches, night sweats, and palpitations. She presented to the emergency department with a blood pressure of 220/110 mmHg and a heart rate of 88 beats per minute. Laboratory testing revealed hypokalemia (2.1 mmol/L). Electrocardiography showed no significant abnormalities. She was admitted for management of hypertension and hypokalemia and further diagnostic evaluation.

**Figure 1:**
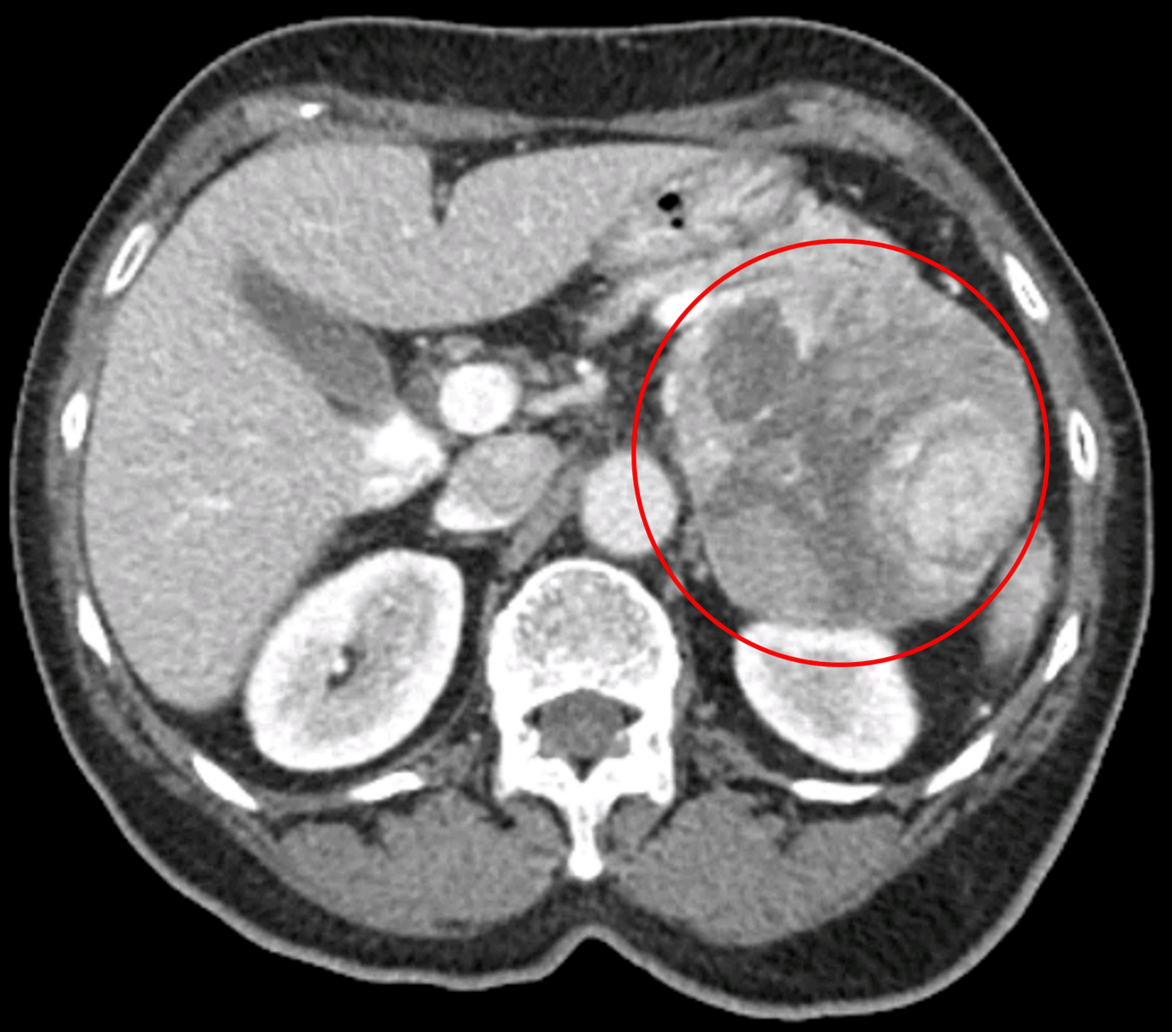
Axial abdominal computed tomography scan showing a 113 mm left adrenal mass (circled)

Abdominal computed tomography (CT) revealed a 113 mm mass arising from the left adrenal gland, displacing the pancreatic tail and spleen (Figure 1). Given the combination of hypertension and hypokalemia, pheochromocytoma was suspected, and a diagnostic workup was initiated. Her relevant laboratory results are summarized in Table 1.

**Table 1:** Relevant laboratory results HbA1c, glycated hemoglobin; TSH, thyroid-stimulating hormone; FT4, free thyroxine; ACTH, adrenocorticotropic Hormone; DHEA, dehydroepiandrosterone.

Laboratory Analytes		Patient Results	Reference Range
Sodium (mmol/L)		140	135-145
Chloride (mmol/L)		96	96-108
Potassium (mmol/L)		2.1	3.5-5.1
HbA1c (%)		8.2	4.3-6.1
TSH (mUI/L)		0.37	0.35-5.5
FT4 (ng/dL)		1.29	0.8-1.76
ACTH (pg/mL)		10.50 (9:20 am)	<46 Morning time
Basal Cortisol (mcg/dL)		40.90	5.27-22.45
Salivary Cortisol (nmol/L)		24.55 (1:30 am)	Nighttime 1.2-12.3
Aldosterone (ng/dL)		4.9	3.47-27.5
Plasma Renin (mUI/L)		8.91	2.8-39.9
Plasmatic Metanephrines (ng/mL/h)		<15	<100
Normetanephrine (ng/mL/h)		52	<216
DHEA (ng/mL)		6.9	0.5-3
Urine Sodium (mEq/24 h)		271	40-220
Urine Potassium (mEq/24 h)		87	25-125
Urine Chlorine (mEq/24 h)		351.9	110-250.0
Urine Aldosterone (mcg/24 h)		2.98	2.84-33.99
Urine Catecholamines (mcg/24 h)	Adrenaline	<5.00	<18.00
	Noradrenaline	48.00	<76
	Dopamine	298.00	<390.00
Urine Metanephrines (mcg/24 h)	Metanephrine	27.00	<341.00
	Normetanephrine	215.00	<444.00

Despite the initial suspicion of pheochromocytoma, the patient’s plasma and urinary metanephrine and catecholamine levels were within reference ranges, making this diagnosis less likely. Given the elevated basal cortisol and dehydroepiandrosterone (DHEA) levels, alternative etiologies were considered, including adrenocortical carcinoma. OACC was specifically hypothesized due to its potential to present with a pheochromocytoma-like syndrome.

**Figure 2:**
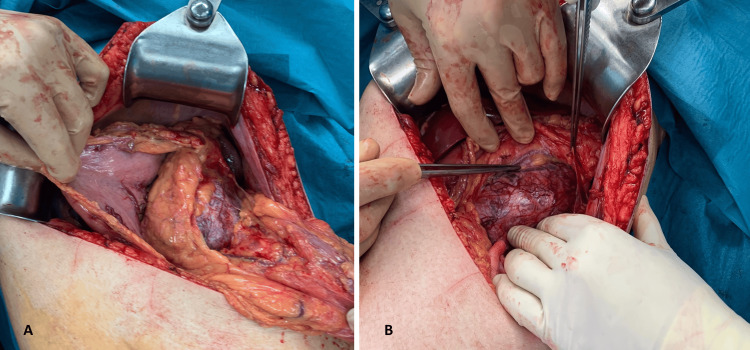
Intraoperative views of the adrenalectomy. (A) Initial incision through the peritoneal fascia exposing the tumor. (B) Mobilization of the mass within the retroperitoneal space

**Figure 3:**
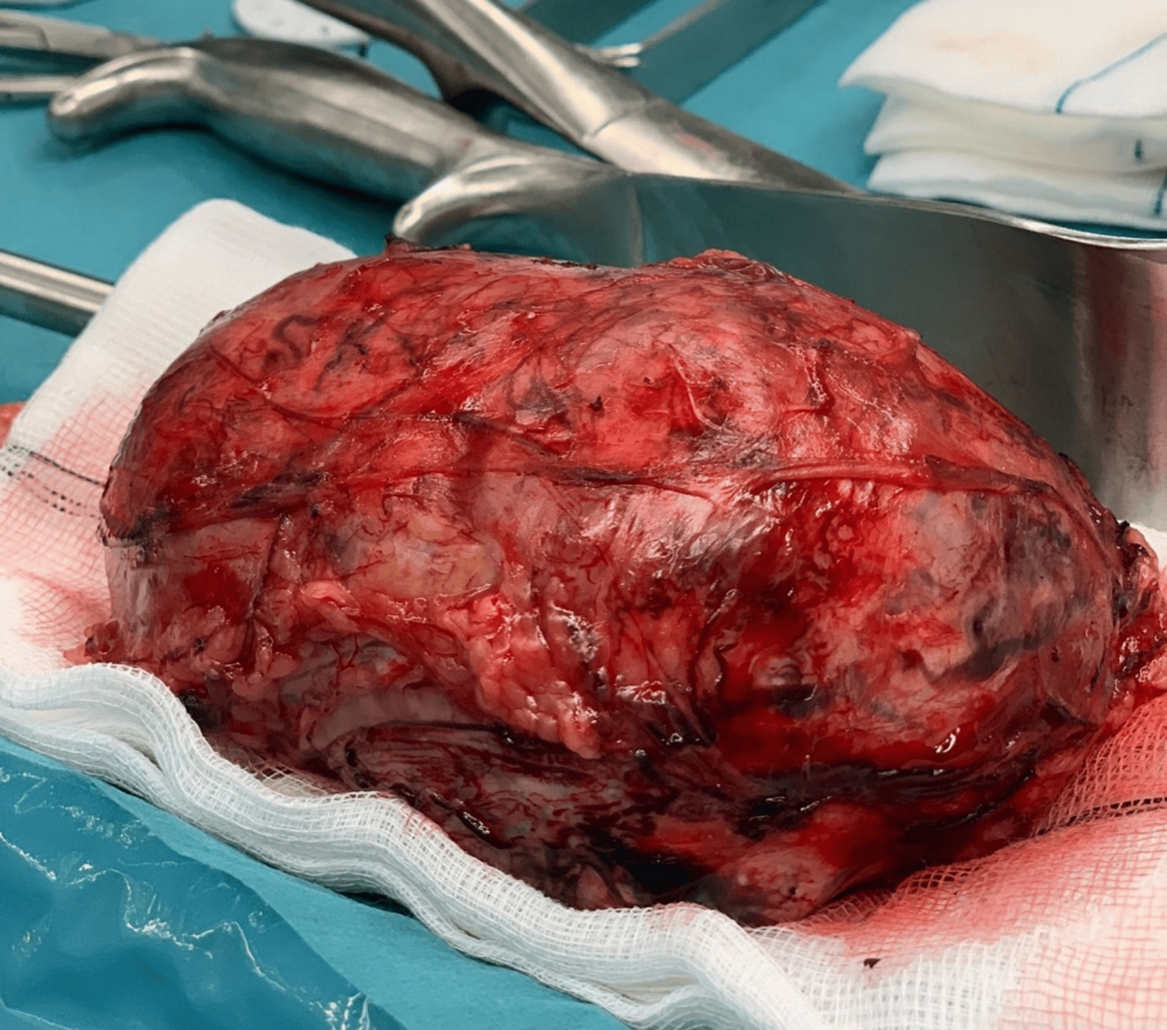
Resected adrenal tumor measuring 123 × 90 × 60 mm and weighing 355 g

A multidisciplinary team meeting involving Endocrinology, General Surgery, and Internal Medicine determined that adrenalectomy via laparotomy was indicated to obtain a definitive histopathologic diagnosis and address the underlying cause of the patient’s symptoms. Intraoperative findings are shown in Figure 2. A 355 g adrenal mass measuring 123 × 90 × 60 mm was excised (Figure 3).

**Figure 4:**
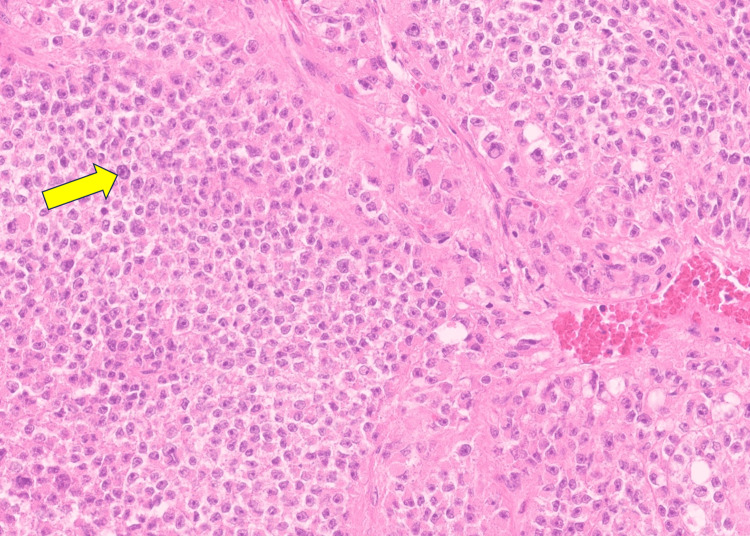
Hematoxylin‑ and eosin‑stained section demonstrating oncocytic tumor cells (arrow denotes Hurthle cell) with granular eosinophilic cytoplasm (400x magnification)

Histopathologic examination (Figure 4) demonstrated a neoplasm composed of cells with abundant eosinophilic cytoplasm and prominent round nucleoli, consistent with oncocytic morphology and Fuhrman nuclear grade IV. Scattered cells exhibited marked nuclear pleomorphism. The tumor architecture showed nests and trabeculae, with disruption of the normal reticulin framework. A total of 36 mitoses per 50 HPFs were observed, including atypical forms. The Ki-67 proliferation index was 10%. Extensive areas of necrosis and foci of venous invasion were identified. Immunohistochemical staining revealed the following profile: calretinin positive, Melan-A weakly positive, synaptophysin positive, and inhibin weakly positive. Adjacent normal adrenal tissue was present, and surgical margins were uninvolved. Based on the LWB criteria, the tumor met all three major criteria (mitotic index >5/50 HPFs, atypical mitoses, venous invasion) and three minor criteria (size >100 mm, weight >200 g, and necrosis), confirming the diagnosis of OACC.

Postoperatively, the patient experienced an uneventful recovery. She was started on steroid replacement therapy and achieved adequate blood pressure control with nebivolol 5 mg daily, spironolactone 25 mg daily, and nifedipine 30 mg twice daily. Additionally, she was diagnosed with type 2 diabetes and initiated on metformin 700 mg twice daily. At the time of discharge, she remained clinically stable, with controlled blood pressure and close outpatient follow-up arranged. She was subsequently initiated on mitotane therapy in accordance with the current clinical guidelines. At the six-month follow-up, CT imaging showed no evidence of recurrence. Mitotane level was 16.7 mg/L, DHEA had normalized to 2.1 ng/mL, and basal cortisol was 17.54 mcg/dL, supporting the conclusion that the tumor was hormonally active and the source of the patient’s clinical presentation.

## Discussion

Oncocytes were first described by Hamperl in 1962 as polyhedral cells with voluminous, finely granular eosinophilic cytoplasm [7]. These cells are characterized by the accumulation of numerous mitochondria, which accounts for their abundant eosinophilic granular cytoplasm [8]. Although the precise mechanisms underlying oncocytosis remain unclear, two main theories have been proposed: one suggests that mitochondrial proliferation results from genetic mutations, while the other attributes it to epigenetic changes secondary to cellular hypoxia [9].

Oncocytic neoplasms most commonly arise in the left adrenal cortex [10], with only a single case reported to extend into the renal medulla [11]. In rare instances, ectopic tumors have been identified in the retroperitoneum, cecum, liver, kidney, and lumbar spine [12-15]. These tumors exhibit a female predominance (female-to-male ratio of 1.9:1) and can occur across all age groups, with peak incidence in the fourth and fifth decades of life [4].

Most oncocytic adrenocortical tumors are nonfunctional and are often discovered incidentally (referred to as incidentalomas). However, lesions larger than 4 cm are generally considered potentially malignant and warrant surgical evaluation. Approximately 30% of oncocytic adrenal neoplasms are classified as malignant based on histopathologic criteria [4]. Hormonal hypersecretion is observed in approximately 31.5% of cases, with presentations mimicking Cushing syndrome or pheochromocytoma [16].

Imaging modalities such as CT and magnetic resonance imaging have limited specificity in diagnosing OACC, as these tumors are often radiologically indistinguishable from other adrenal malignancies. Typically, oncocytic carcinomas appear as large (i.e., with an average size of approximately 110 mm), heterogeneously enhancing masses with possible fibrous encapsulation on imaging studies [1].

Macroscopically, these tumors are well circumscribed and encapsulated, often demonstrating areas of hemorrhage and necrosis. Histologically, they consist of large oncocytic cells with granular eosinophilic cytoplasm, reflecting mitochondrial accumulation. Immunohistochemical staining is usually positive for vimentin, calretinin, α-calretinin, α-inhibin, and Melan-A, and negative for S100 and chromogranin [17].

Given their potential for hormonal secretion and malignancy, surgical resection is the primary treatment. Adrenalectomy is typically performed via laparotomy; however, laparoscopic approaches are increasingly considered for tumors smaller than 6 cm, provided complete excision without capsular disruption can be ensured [10].

Adjuvant therapy with mitotane remains the standard of care due to its demonstrated effect in reducing tumor recurrence. One study reported a 50% reduction in recurrence rates with mitotane treatment [18]. Therapeutic drug monitoring is crucial for maintaining serum levels within the therapeutic range. Despite its known adverse effects (including anorexia, nausea, somnolence, lethargy, and electroencephalogram abnormalities), mitotane continues to be the most effective adjuvant therapy for patients with OACC.

## Conclusions

OACC is a rare and diagnostically challenging malignancy. Surgical excision remains the cornerstone of treatment, with adjuvant mitotane therapy considered to reduce the risk of recurrence. As illustrated by this case, OACC should be considered in patients presenting with uncontrolled hypertension and elevated basal cortisol and DHEA levels. The application of the LWB criteria aided in distinguishing OACC from other adrenal neoplasms, given its specificity for this tumor type. The patient’s favorable response to adjuvant mitotane therapy, with no evidence of recurrence at the six-month follow-up, supports its clinical utility in reducing relapse risk. By sharing this case, we aim to contribute to the growing body of literature and enhance the understanding of the diagnosis, management, and prognosis of this uncommon tumor.
